# Soil Warming Accelerates Biogeochemical Silica Cycling in a Temperate Forest

**DOI:** 10.3389/fpls.2019.01097

**Published:** 2019-09-11

**Authors:** Jonathan Gewirtzman, Jianwu Tang, Jerry M. Melillo, William J. Werner, Andrew C. Kurtz, Robinson W. Fulweiler, Joanna C. Carey

**Affiliations:** ^1^The Ecosystems Center, Marine Biological Laboratory, Woods Hole, MA, United States; ^2^Institute at Brown for Environment and Society, Brown University, Providence, RI, United States; ^3^Department of Biology, Boston University, Boston, MA, United States; ^4^Department of Earth and Environment, Boston University, Boston, MA, United States; ^5^Division of Math and Science, Babson College, Babson Park, MA, United States

**Keywords:** silica, climate change, soil, warming, phytoliths, plants, biogeochemistry

## Abstract

Biological cycling of silica plays an important role in terrestrial primary production. Soil warming stemming from climate change can alter the cycling of elements, such as carbon and nitrogen, in forested ecosystems. However, the effects of soil warming on the biogeochemical cycle of silica in forested ecosystems remain unexplored. Here we examine long-term forest silica cycling under ambient and warmed conditions over a 15-year period of experimental soil warming at Harvard Forest (Petersham, MA). Specifically, we measured silica concentrations in organic and mineral soils, and in the foliage and litter of two dominant species (*Acer rubrum* and *Quercus rubra*), in a large (30 × 30 m) heated plot and an adjacent control plot (30 × 30 m). In 2016, we also examined effects of heating on dissolved silica in the soil solution, and conducted a litter decomposition experiment using four tree species *(Acer rubrum, Quercus rubra, Betula lenta, Tsuga canadensis)* to examine effects of warming on the release of biogenic silica (BSi) from plants to soils. We find that tree foliage maintained constant silica concentrations in the control and warmed plots, which, coupled with productivity enhancements under warming, led to an increase in total plant silica uptake. We also find that warming drove an acceleration in the release of silica from decaying litter in three of the four species we examined, and a substantial increase in the silica dissolved in soil solution. However, we observe no changes in soil BSi stocks with warming. Together, our data indicate that warming increases the magnitude of silica uptake by vegetation and accelerates the internal cycling of silica in in temperate forests, with possible, and yet unresolved, effects on the delivery of silica from terrestrial to marine systems.

## Introduction

Climate change is expected to cause pervasive alterations to ecosystem structures, functions, and processes in the coming decades (Grimm et al., 2013; Scheffers et al., 2016), resulting in complex and varied feedbacks to the climate system (Shaver et al., 2000; Field et al., 2007; IPCC, 2013). Climactic warming affects ecosystem processes, such as carbon storage in plants (Melillo et al., 1993; Chapin et al., 1995; Lin et al., 2010; Melillo et al., 2011) and carbon release from soils (Peterjohn et al., 1994; Rustad et al., 2001; Melillo et al., 2002; Carey et al., 2016; Melillo et al., 2017). Much research has been conducted regarding the interaction between climate change and the biogeochemical cycling of elements, such as nitrogen and carbon (Rustad et al., 2001; Melillo et al., 2011; Bernal et al., 2012; Butler et al., 2012), and the plant stoichiometry of carbon, nitrogen, and phosphorus (An et al., 2005; Elser et al., 2010; Dijkstra et al., 2012; Sardans et al., 2012). However, less attention has been paid to the effects of soil warming on biogeochemical cycling of other globally important elements, such as silicon.

Silica [silicon dioxide (SiO_2_)] is the most abundant compound in the Earth’s crust (Brown and Mussett, 1981) and soils (Epstein, 1994; Tréguer and De La Rocha, 2013). Silica and carbon are coupled in terrestrial and marine ecosystems through processes such as mineral silicate weathering, phytolith-occluded carbon storage in soils, and primary production by terrestrial and marine silica-accumulating organisms (Street‐Perrott and Barker, 2008; Carey and Fulweiler, 2012a; Song et al., 2012). Therefore, understanding the impacts of climactic warming and environmental change on silica cycling is important for modeling and predicting future global carbon cycling.

Over geological timescales, the weathering of silicate minerals consumes carbon dioxide (CO_2_), making the process a significant control on atmospheric CO_2_ and planetary climate (Urey, 1952; Berner et al., 1983; Berner, 1990; Street‐Perrott and Barker, 2008). Mineral silicate weathering is driven by complex interactions between the climate and lithosphere (Bluth and Kump, 1994; Hilley and Porder, 2008), as well as the biosphere (Drever, 1994; Berner, 1997; Conley, 2002; Derry et al., 2005). The terrestrial biosphere also acts as a filter for weathering-derived silica before its eventual export to oceans (Struyf and Conley, 2012). Plants take up silica as dissolved silicic acid (DSi, H_4_SiO_4_), the dominant form of SiS in soil solutions (Epstein, 1994). They convert DSi to biogenic silica (BSi, hydrated SiO_2_) whereupon it is deposited in siliceous structures, primarily phytoliths, in the plant biomass (Sangster and Parry, 1976; Canny, 1990; Fu et al., 2002; Trembath-Reichert et al., 2015). Silica concentrations in terrestrial vegetation vary widely, with some plants taking up silica in greater proportion than macronutrients like nitrogen or phosphorus (Epstein, 1994; Hodson et al., 2005). Carey and Fulweiler (2012a) estimate that active Si-accumulating plants are responsible for more than half of terrestrial net primary production (NPP), linking atmospheric CO_2_ and terrestrial silica cycling on biological timescales.

BSi accumulation in plants and soil has been shown to regulate the magnitude and phenology of forest silica cycling and watershed export in some systems (Meunier et al., 1999; Fulweiler and Nixon, 2005; Struyf et al., 2009a; Clymans et al., 2016). Plants return BSi to soil chiefly as fine litterfall (Bartoli, 1983; Struyf and Conley, 2012; Clymans et al., 2016), and phytoliths from this pool can accumulate throughout the topsoil (Struyf et al., 2009b; Cornelis et al., 2011). While many factors, such as pH and species differences in phytolith structure, can affect BSi dissolution rates in soil (Wilding and Drees, 1974; Fraysse et al., 2009), BSi is 7–20 times more soluble than mineral silicates in soils, often resulting in efficient recycling to DSi (Farmer et al., 2005; Fraysse et al., 2006; Fraysse et al., 2009; Struyf et al., 2009a; Cornelis et al., 2011). Thus, the soil BSi pool can be an important supply of DSi to soil solution and streams in diverse ecosystems, particularly high-Si accumulating systems, such as grasslands and deciduous forests, and highly weathered (Si-depleted) systems, such as tropical forests (Derry et al., 2005; Gérard et al., 2008; Struyf et al., 2009a; Lugolobi et al., 2010; Struyf and Conley, 2012). In a North American temperate deciduous forest watershed, Clymans et al. (2016) found that a minimum of 50% of annual soil DSi production derives from BSi dissolution, with 98% of that supply deriving from fresh leaf litter.

Terrestrial systems supply ∼78% of annual silica inputs to oceans (Tréguer and De La Rocha, 2013), where silica is essential for a wide range of species including diatoms, which are responsible for approximately half of marine primary production (Tréguer et al., 1995; Rousseaux and Gregg, 2013). Because diatom productivity can be limited or co-limited by silica availability (Nelson and Treguer, 1992; Leynaert et al., 2001; Brzezinski et al., 2008), the magnitude of silica delivery from terrestrial to marine systems can impact marine and global NPP.

Anthropogenic perturbations, such as deforestation, urbanization, and agriculture, are known to alter terrestrial silica biogeochemistry (Conley et al., 2008; Laruelle et al., 2009; Vandevenne et al., 2012; Carey and Fulweiler, 2012b; Carey and Fulweiler, 2016; Unzué-Belmonte et al., 2017). However, the role of climate change on terrestrial silica biogeochemistry remains less well known. Recently, experimental CO_2_ and nitrogen enrichment was shown to increase forest silica uptake (Fulweiler et al., 2015), while experimental snowpack reduction and induced soil freezing was shown to impede plant silica uptake capacity (Maguire et al., 2017). Still, while there has been much research and discussion of the impact of temperature on long-term silica geochemistry (Brady, 1991; Velbel, 1993; Brady and Carroll, 1994; Turner et al., 2010), there has been no study to date addressing the impact of temperature on terrestrial silica biogeochemistry.

To explore the effects of soil warming on terrestrial silica cycling, we analyzed BSi in soils, foliage, litter, and soil solution samples taken over 15 years of a long-term soil warming experiment (Melillo et al., 2011). We also conducted a litter decomposition experiment to explore dynamics of plant silica release under ambient and warmed conditions. We hypothesized that soil warming would increase tree silica uptake as a result of increased productivity. We also hypothesized that soil warming would increase decay rates thereby accelerating the release of silica from decomposing litter. Finally, we hypothesized that these changes would increase forest silica recycling with minor, if any, net effect on soil BSi storage over the timespan of the study.

## Materials and Methods

### Site Description

The Barre Woods soil warming experiment is located in an even-aged, mixed deciduous stand at Harvard Forest in Petersham, MA (42° 28′ N, 72° 10′ W). Tree species composition is dominated by oak (*Quercus rubra and Quercus velutina*), red maple (*Acer rubrum*), and American ash (*Fraxinus americana*), comprising 42%, 29%, and 11% of basal area, respectively (Melillo et al., 2011; Melillo et al., 2017). The site was historically used for pastureland or low-intensity agriculture, and then came to be dominated by white pines in the first half of the 20th century. In 1938, a hurricane destroyed much of the stand, which was then left to regrow to its current state (Melillo et al., 2011). Soils at the study site are of the Canton series, with O horizon pH of 5.2 and subsurface mineral horizon pH of 5.5 (Melillo et al., 2011). Mean weekly air temperature at the site varies from a high of approximately 20°C in July to a low of approximately −6°C in January, and mean annual precipitation is approximately 1080 mm, distributed evenly throughout the year (Melillo et al., 2011).

### Soil Warming Experiment

Complete descriptions of the warming experiment methods have been previously published (Melillo et al., 2011) and are detailed in the Harvard Forest Data Archive (Melillo et al., 2017). Briefly, in 2001, heating cables were buried in a 30 × 30-m area at 10-cm depth with 20-cm spacing. An adjacent 900 m^2^ plot serves as the control area, separated from the heated plot by a 5-m buffer. Starting in 2003, the heating cables were cycled on and off to maintain soil temperatures in the heated plot elevated at 5°C above control plot soil temperatures. Soil warming continued for the duration of the measurements and sample collection in this study.

### Soil and Vegetation Sampling

Soils were sampled to 10 cm in the control and heated plots, divided visually into organic and mineral horizons, sieved to 2 mm, and dried. We subsampled soils for silica analysis from a pre-treatment year (2002), and from three other years during the study (2005, 2010, and 2016). We analyzed three samples per layer in each plot for each of the 4 years (n = 48). We subsampled only from cores taken early in the growing season.

Foliage (green leaves) was sampled by shotgun during the summer between June and August. Four to five trees from each plot were sampled, preferentially selecting sunlit leaves, which were then bulked together, dried, homogenized, and milled. Each year’s foliage sample, thus, represent a homogenized sample for a given species and plot. We measured triplicate subsamples of samples from 7 years between 2003 and 2016 (approximately every 2 years during the course of the study, n = 28). We report green leaf BSi values as the mean of those three subsamples.

Leaf litter was collected in baskets installed in each plot. Wire baskets dispersed throughout the plots were used from 2003 to 2006; thereafter, laundry baskets clustered in the center of the plots were used for litter collections. The litter was collected regularly from each basket during the fall, dried, and sorted by species. Fresh leaf litter samples were kept separate by collection basket (n = 3 per plot) for all years except 2008; in 2008, samples were bulked across baskets by species and treatment. For all years, the bulked litter samples were homogenized and milled. We analyzed subsamples from the same 7 years as the green foliage (n = 84). For all years, except 2008, we analyzed samples from each of three collection baskets for each species–treatment. For 2008, we analyzed three subsamples of the bulked sample from each species × treatment and calculated a single mean BSi value for that species × plot × year.

### Soil Solution and Stream Water Sampling

In 2016, we collected soil water samples using lysimeters previously installed in the plots. Six porous cup high-tension lysimeters were installed in each plot at a depth of 50 cm and evacuated. We sampled at approximately monthly intervals from May to December 2016. Lysimeters were evacuated to ∼380 mm Hg the day before sampling. We retrieved as many samples as possible on each sampling occasion; however, we often recovered fewer than six samples per plot per sampling interval due to low soil water content. Soil water was filtered through a polypropylene syringe using a 0.45-μm nitrocellulose filter immediately upon retrieval. We retrieved and analyzed a total of 40 samples (21 from the heated plot and 19 from the control plot).

On each day that we sampled from lysimeters, we also sampled stream water from a nearby stream in the Prospect Hill tract of Harvard Forest, Bigelow Brook, at the Lower Pipe stream gauge. Three stream samples were collected at each of the seven sampling time points (n = 21), within an hour after lysimeter sample collection. To do this, 60 mL of water was drawn in a polypropylene syringe and filtered using 0.4-µm polycarbonate filters to isolate suspended silica. We measured DSi in the filtered water samples and suspended BSi captured on the filters to estimate total stream water silica. All lysimeter, stream water, and filter samples were kept refrigerated until analysis.

### Litter Decomposition Experiment

In 2016, we also conducted a litter decomposition experiment where we collected litter from four common species at the site: *Quercus rubra* (red oak), *Acer rubrum* (red maple), *Betula* spp. (mixed birch; mostly *Betula lenta*), and, *Tsuga canadensis* (eastern hemlock). We chose these species to align with an earlier wood decomposition study in the same soil warming plots (Berbeco et al., 2012). We collected litter from the forest floor, outside of the study plots, in October 2015. We homogenized litter by species, rinsing with deionized water to remove soil, and placed them in a drying oven overnight at 60°C. We placed approximately 5 g of litter of single species into 20 × 20 cm bags made of 5-mm fiberglass mesh. The bags were closed on all sides with an impulse sealer and tagged with unique plastic identification tags.

We placed 21 bags of each of the four species in transects in each of the two plots (21 × 4 × 2 = 168 bags total) during May 2016. We placed litterbags between the Oi and Oe horizons, with at least 10-cm spacing between bags. We tied the bags to plastic stakes with nylon string for future retrieval. Subsamples of the four litter types were used and was kept in the laboratory for analysis of their initial chemical composition. We collected three litterbags from each species at each plot at approximately monthly intervals from May through December of 2016 (3 replicates × 4 species × 2 plots = 24 bags). Upon collection, we gently rinsed and dried bags at 60°C to constant mass. We then ground the contents of bags using a Wiley mill for subsequent silica analysis.

### Chemical Analysis

We measured BSi concentrations in all of the aforementioned samples using a wet alkaline chemical extraction in a 1% Na_2_CO_3_ solution (DeMaster, 1981; Conley and Schelske, 2001). Duplicate subsamples were taken from each sample and weighed to approximately 30 mg. We digested samples in flat-bottomed polyethylene bottles in a shaking water bath at 85°C and 100 rpm. For leaf and litter samples, a single aliquot was taken from each digestion bottle after 4 h for analysis. No separation into mineral and amorphous/biogenic fractions was necessary given that all silica contained in those samples is by definition biogenic. For soils and stream water filters, the fraction of DSi released from BSi was determined by time-course extraction (aliquots taken at 3, 4, and 5 h), followed by a linear extrapolation to the intercept (DeMaster, 1981; Saccone et al., 2007). For all digested samples, aliquots taken were of 1 mL and were neutralized in 9 mL of 0.021 M HCl.

All extracted samples, as well as soil water and stream water, were analyzed for DSi using the molybdenum blue colorimetric method (Strickland and Parsons, 1968). Standards made of sodium hexafluorosilicate, as well as external standards, were used throughout the analysis to check accuracy. All errors between duplicate samples were less than 5%. Digestions were conducted at Brown University (Providence, RI), and colorimetric analyses were conducted at Boston University (Boston, MA) using a Seal AA3 flow injection autoanalyzer.

### Statistical Analysis

We applied a pretreatment correction factor to our soil BSi data, following Melillo et al. (2011). The pretreatment correction factor scales the initial heated data to equal the control. Pretreatment samples were only available for soil, so only the soil data are presented with this correction.

All statistical analyses were conducted using R Version 3.4.4 (R Core Team, 2018). Soil data were analyzed using linear mixed-effects models in the “nlme” R package (Pinheiro et al., 2018), with year, layer, and treatment as fixed effects and subplot as a random effect. Green leaf BSi was analyzed using linear regression with single annual foliar concentrations as a function of sampling month, species, and treatment. Leaf litter BSi was analyzed using linear mixed-effect models with year, species, and treatment as fixed effects, and collection basket as random effect. Random effects were nested within litter basket to account for repeated measures and autocorrelation.

Using public litter mass data from the soil warming experiment (Melillo et al., 2017) and our measured BSi concentrations, we calculated annual litterfall masses and litterfall BSi fluxes. We fit the data to a linear mixed effects model, with litter mass per area (log-transformed) as product of species, treatment, year, and collection basket type, with specific basket location as a random effect. We excluded the data from 2015, when a major summertime hailstorm caused widespread defoliation prior to autumn leaf senescence and abscission.

For the decomposition experiment data, we calculated the percent of initial mass remaining for each litter bag as the quotient of final dry mass and initial dry mass. We then used a single exponential model to calculate a decay constant (Olson, 1963; Berbeco et al., 2012):

$$\text{k}=[\text{ln}({\text{M}}_{\text{0}}-\text{ln}({\text{M}}_{\text{t}})]/t$$

where *k* is the decay constant, M_t_ is the percent of remaining biomass at time, *t*, and *M*_0_ is 100%. We also calculated the estimated time to decompose 95% of matter (Berbeco et al., 2012), using the equation:

$${\text{t}}_{0.95}=-\text{ln}(0.05)/\text{k}$$

We analyzed the effects of species and treatment on mass loss, elemental composition, and elemental ratios using linear regression, with each response as a function of species, treatment, time, and interactions among these variables. Decay constants were regressed against species, treatment, and interactions. Data were rank-transformed prior to analysis to meet assumptions regarding homoscedasticity.

Stream and porewater dissolved silica were analyzed using a two-way ANOVA with sampling date and DSi pool as the main effects, followed by a *post hoc* Tukey’s HSD test. Data were also rank-transformed prior to analysis.

We evaluated the normality of model residuals using visual inspection and Shapiro-Wilk normality tests. Significance for all statistical tests was judged using an alpha of 0.05. We report silica concentrations by percent dry weight as SiO_2_ (%BSi) unless otherwise specified, and all errors reported in text and figures are standard errors of the mean.

## Results

### Soil BSi

**Figure 1:**
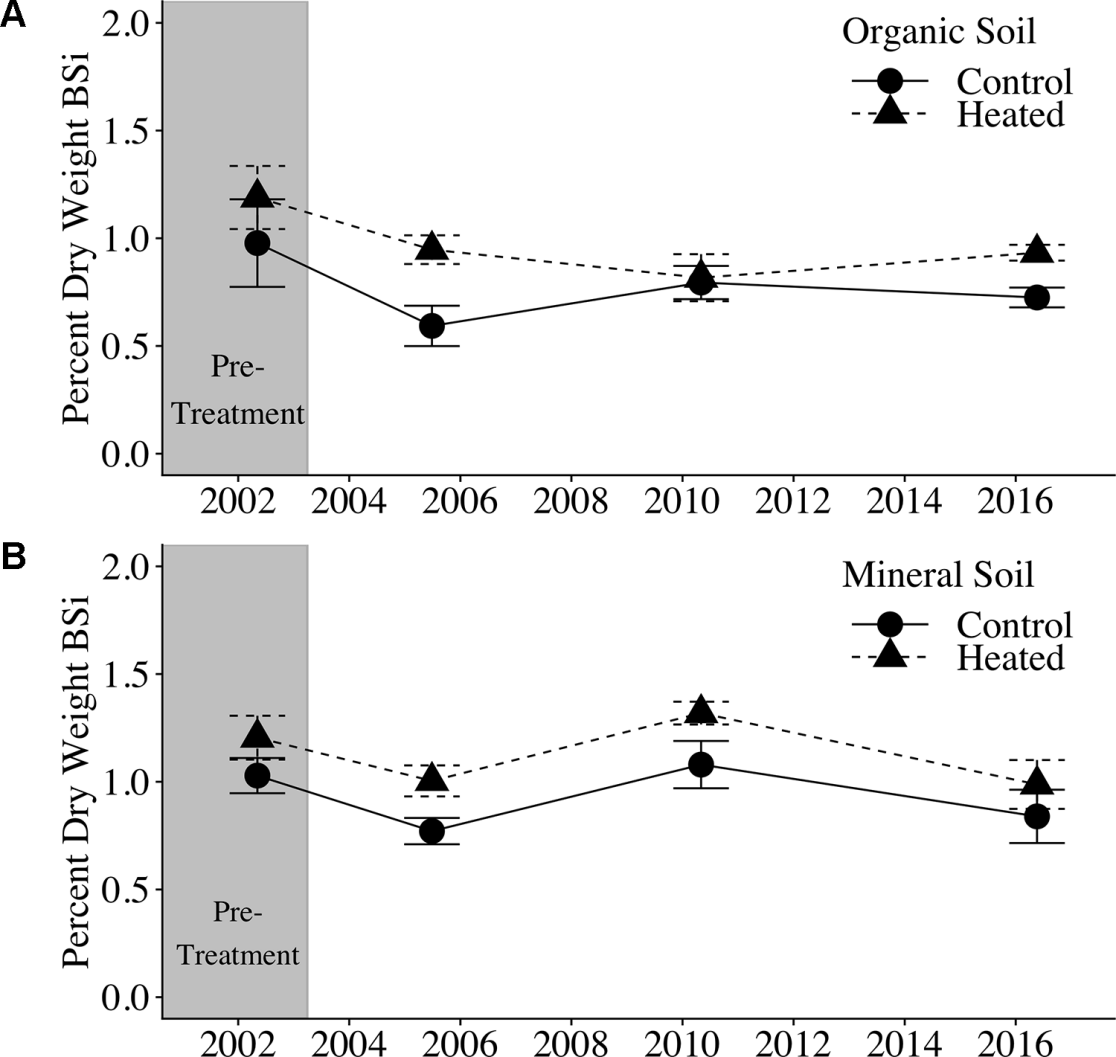
Organic and mineral soil BSi concentrations. Organic soil percent BSi **(A)** and mineral soil percent BSi **(B)** are shown for the pre-treatment year (2002) and three treatment years. Error bars represent standard errors of the mean.

Soil BSi concentration across all samples (**Figure 1**) averaged 0.95% ± 0.34%, and was higher in the organic layer (1.03% ± 0.04%) than in the mineral layer (0.87% ± 0.05%).

We applied a pretreatment correction factor to our soil BSi data, following Melillo et al. (2011), to account for the fact that soil BSi concentrations were higher in the heated plot compared to the control plot before treatment began (17% higher in organic soil and 21% higher in mineral soil; uncorrected data available in **Supplementary Tables 1** and **2**). The pretreatment correction factor scales the initial heated data to equal the control, so that we can appropriately compare control and treated samples.

Pretreatment-corrected soil BSi concentrations (**Table 1**) varied significantly between organic and mineral layers (p = 0.001), but did not vary between control and heated treatments (p = 0.485) or with warming duration (p = 0.623).

**Table 1:** Soil BSi concentrations by year.

Layer	Plot	% Dry wt BSi (2005)	% Dry wt BSi (2010)	% Dry wt BSi (2015)
Organic	Control	0.77 ± 0.06	1.08 ± 0.11	0.84 ± 0.12
	Heated	0.86 ± 0.06	1.13 ± 0.05	0.84 ± 0.10
Mineral	Control	0.59 ± 0.09	0.79 ± 0.08	0.73 ± 0.05
	Heated	0.78 ± 0.05	0.67 ± 0.09	0.77 ± 0.03

BSi, biogenic silica. BSi concentrations are reported as percent dry weight BSi (SiO_2_). Heated plot concentrations are pretreatment-corrected.

Mean bulk density at the Barre Woods site was previously reported to be 0.37 g cm^−3^ in the organic layer and 0.78 g cm^−3^ in the mineral layer, with mean organic layer depth of 1.4 cm (Melillo et al., 2011). Using these values and treatment mean BSi concentrations, we calculated BSi storage in the top 10 cm of soil for the control and heated plots (**Table 2**).

**Table 2:** Soil BSi stocks. Soil BSi stocks were calculated for the top 10 cm in each plot, and the data reported here are means across all samples analyzed from all years during experimental treatment. Heated plot values are pretreatment-corrected.

Layer	Treatment	BSi (kg ha ^-1^), pre-treatment corrected
Organic	Control	464.44 ± 35.83
	Heated	488.01 ± 30.12
Mineral	Control	4723.97 ± 320.07
	Heated	4957.93 ± 240.47

### Foliar and Litter BSi

Silica concentrations were significantly different in foliage vs. leaf litter (p < 0.001), with litter having consistently higher BSi concentrations than green leaves. Green leaf BSi concentrations varied significantly by species (p < 0.001; **Table 3**) and by sampling month (p < 0.001). However, green leaf BSi concentrations did not vary between years (p = 0.817) nor with warming treatment (p = 0.149).

**Table 3:** Foliar and litter BSi concentrations. The values reported are mean concentrations of BSi (percent dry weight) across all samples analyzed (all sampled during experimental treatment, from 7 years between 2003 and 2016; further detailed in methods above).

Sample Type	Species	Treatment	% BSi as dry weight	n
Green Leaf	Red Maple	Control	1.16 ± 0.05	7
		Heated	1.29 ± 0.06	7
	Red Oak	Control	0.37 ± 0.01	7
		Heated	0.38 ± 0.01	7
Leaf Litter	Red Maple	Control	1.86 ± 0.06	21
		Heated	1.78 ± 0.07	20
	Red Oak	Control	0.55 ± 0.01	21
		Heated	0.57 ± 0.01	21

**Figure 2:**
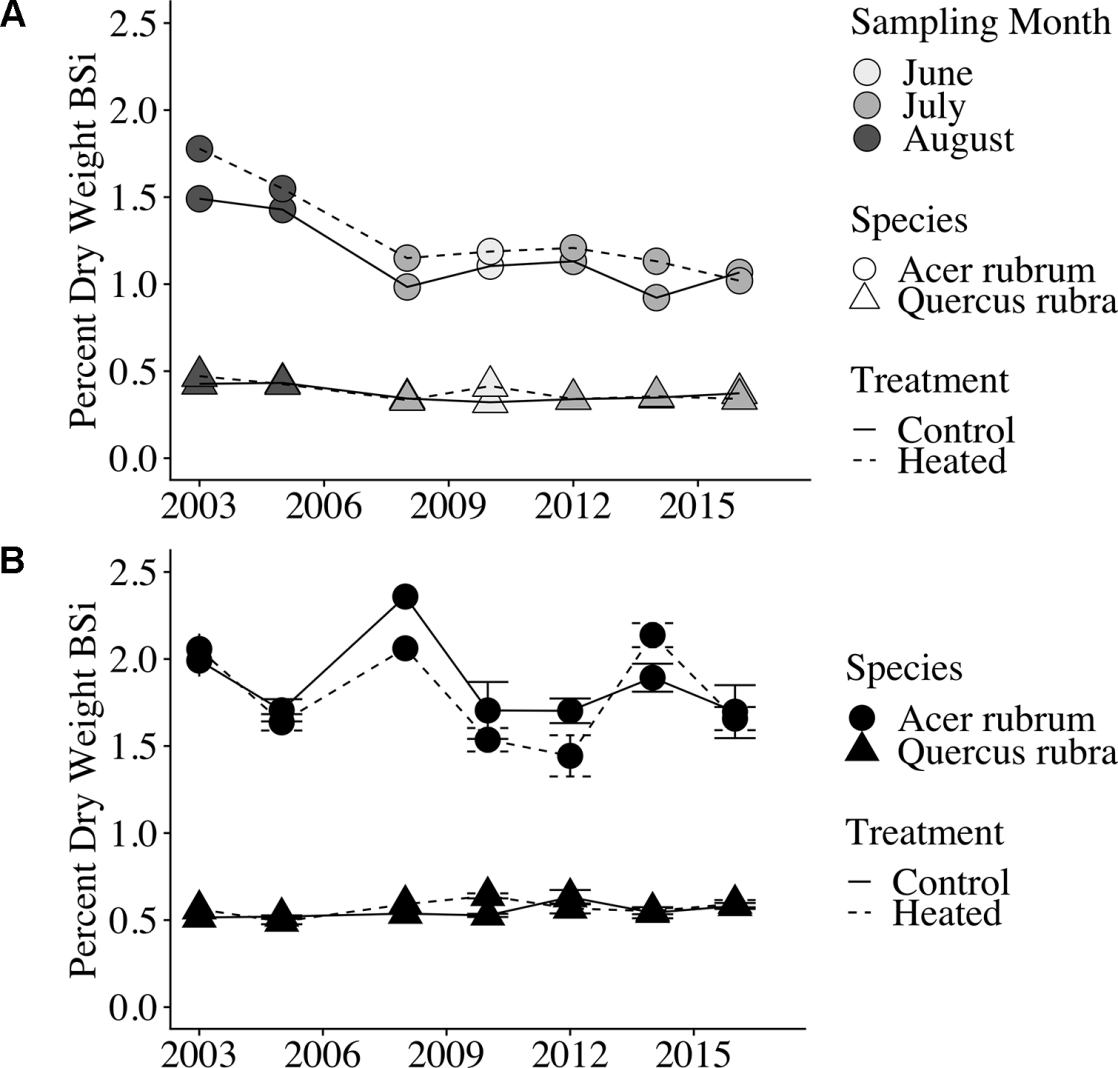
Foliar and litter BSi concentrations over time. **(A)** Foliar (green leaf) BSi concentrations. Point shape indicates species, line type indicates treatment, and point weight indicates the month of sample collection. Samples were collected during August in the first 2 years analyzed; samples were collected in June or July in all subsequent years analyzed. Error bars are not shown because only a single bulked sample was available per plot × year. **(B)** Leaf litter silica concentrations (percent dry weight BSi) are shown for each of the 7 years of analyzed samples; error bars indicate standard error of the mean. All samples were bulked across the duration of litterfall for the given year.

Red maple foliar BSi was more than double that of red oak. Red maple foliage contained 1.29% ± 0.06% BSi (by dry mass) in the heated plot and 1.16% ± 0.05% in the control plot. Red oak BSi was 0.38 ± 0.01% in the heated plot and 0.37 ± 0.01% in the control plot. Foliar concentrations varied significantly by sampling month (p < 0.001) and were higher in years when sampling was conducted in the late growing season (August) compared with years where sampling was conducted earlier in the growing season (June or July; **Figure 2A**).

Similar to foliar BSi, litter BSi varied by species (p < 0.001) but did not vary by treatment (p = 0.588). Mean litter BSi concentrations were ∼1.5× higher than green foliar concentrations across years, species, and treatments (**Figure 2B**). As in green foliage, substantially higher BSi concentrations were observed in red maple litter compared with red oak leaf litter.

**Figure 3:**
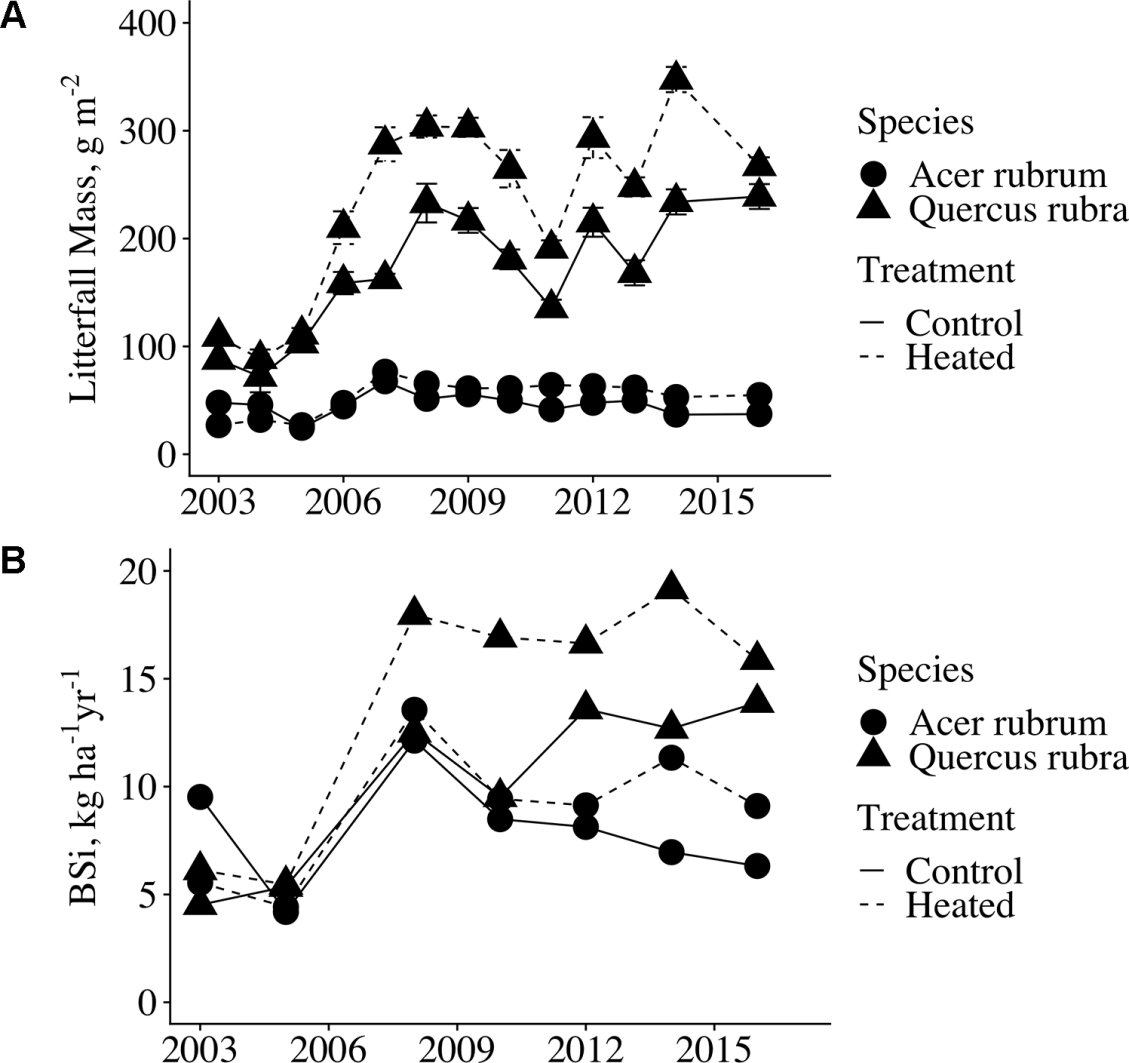
Annual red maple and red oak litterfall mass and litterfall BSi flux. **(A)** The values shown here are mass per area (g m^−2^); mean across all litter baskets per plot. Error bars indicate standard error of the mean. Litterfall was collected from wire baskets dispersed throughout the plots from 2003-2006; thereafter, litterfall was collected in laundry baskets clustered in the centers of the plots. **(B)** Litterfall BSi flux (kg BSi ha^−1^ yr^−1^) was calculated by multiplying mean BSi concentration by mean litterfall mass per area for each year analyzed. Error bars are not shown as measurements were calculated for only the single control plot and the single heated plot.

Although leaf BSi concentrations did not vary between treatments, litterfall production was significantly elevated in the heated plot relative to the control (p < 0.001, **Figure 3A**. Across all years analyzed, mean litter mass for red maple and red oak was elevated 29% from 227 g m^−2^ in the control plot to 293 g m^-2^ in the heated plot. Due to the increase in litter production, leaf litter BSi mass per area was significantly higher in the heated plot compared to the control (p = 0.008; **Figure 3B**). Across all years for which we analyzed samples, mean litter BSi masses were 7.96 kg BSi ha^−1^ in control red maple and 8.91 kg BSi ha^−1^ in heated red maple. In red oak, mean litter BSi masses were 10.30 kg BSi ha ^-1^ in the control and 14.03 kg BSi ha^−1^ in the heated plot.

### Si Dynamics in Litter Decomposition

**Figure 4:**
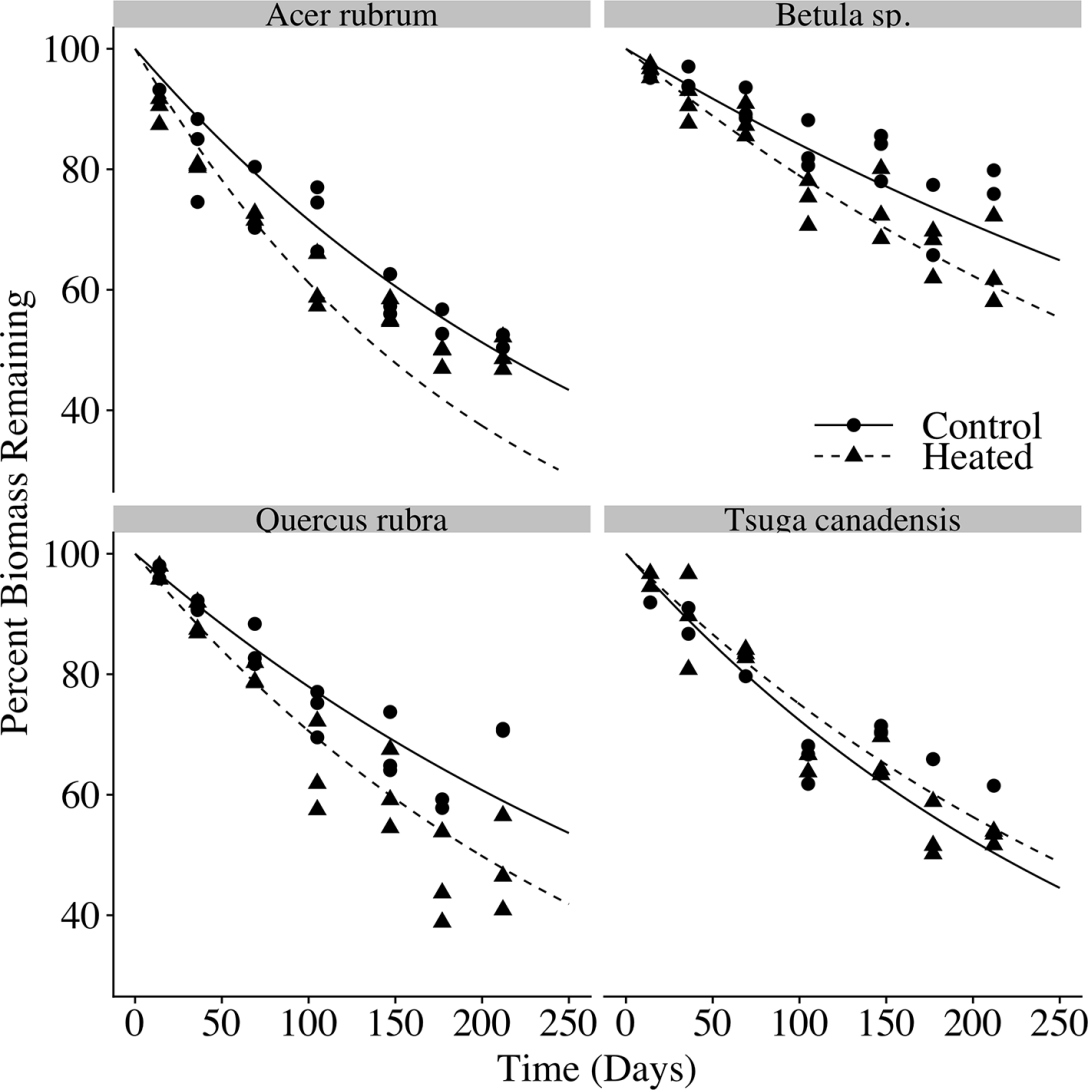
Litterbag mass loss over time. The percent of initial biomass remaining at time of harvest is plotted for each litter bag. Exponential decay curves of the form y = 100e^−kt^ are plotted for the control and heated litterbags for each of the four species, where *k* is the mean decay constant for each species × treatment, and *t* is the number of days deployed.

In each species and treatment, the litterbags decayed to roughly half of their initial masses over the 212 days for which they were allowed to decompose (**Figure 4**). Final litterbag masses were significantly affected by time (p < 0.001), species (p < 0.001) and treatment (p < 0.001), as well as interactions between treatment and time (p = 0.002) and between species and time (p < 0.001).

Across all samples, the modeled time to decay to 95% of original biomass (t95) varied between 1.8 and 5.5 years. Decay constants *k* (**Table 4**) varied according to species (p < 0.001) and treatment (p < 0.001). Heating increased mean decay rates by 47% for red maple, 40% for red oak, and 37% for birch. In contrast, decay rates decreased by 12% in the heated treatment for hemlock; however, it should be noted that several hemlock litterbags were either lost or damaged during retrieval, so the number of replicates was reduced and error was highest among hemlock samples.

**Table 4:** Decay constants and t95 values for litterbag mass. For each set of litterbags (species × treatment), mean decay constant (k), time in years to decompose to 5% of initial mass (t95), and number of litterbags successfully retrieved and analyzed (n). Errors reported are standard error of the mean.

Species	Treatment	k	t95	n
*Acer rubrum*	Control	1.22 ± 0.24	2.05 ± 0.25	17
*Acer rubrum*	Heated	1.79 ± 0.12	1.79 ± 0.1	21
*Betula sp.*	Control	0.63 ± 0.06	5.45 ± 0.47	19
*Betula sp.*	Heated	0.86 ± 0.05	3.7 ± 0.22	21
*Quercus rubra*	Control	0.91 ± 0.05	3.51 ± 0.23	19
*Quercus rubra*	Heated	1.27 ± 0.08	2.64 ± 0.24	21
*Tsuga candadensis*	Control	1.18 ± 0.12	2.78 ± 0.22	13
*Tsuga candadensis*	Heated	1.05 ± 0.15	2.66 ± 0.39	21

**Figure 5:**
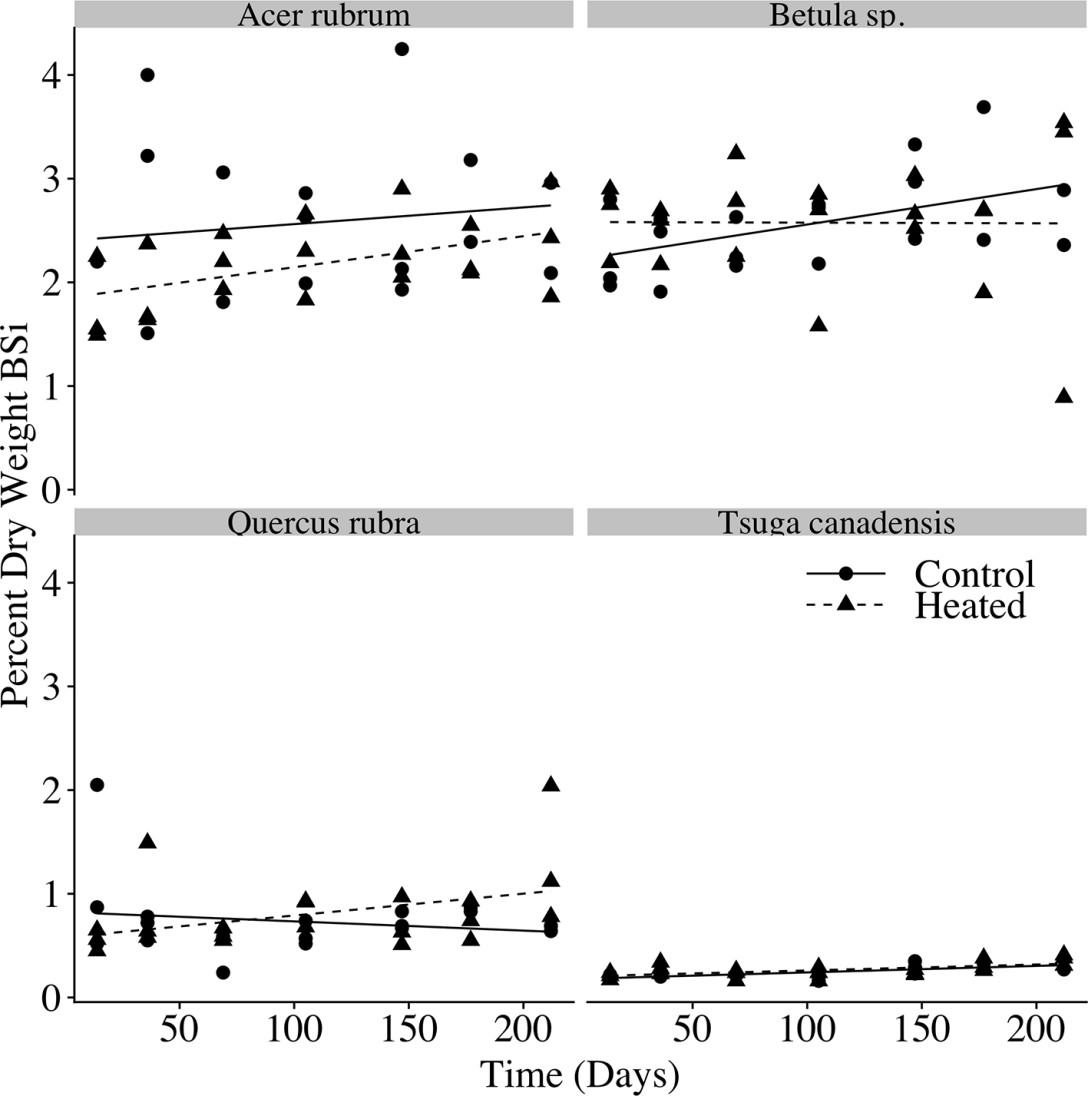
Litterbag BSi concentration over time. Biogenic silica (BSi) concentration (percent dry weight BSi) for each litterbag at time of harvest. Linear regression models are plotted for the control and heated litterbags of each species.

The percent of litter composed of BSi (**Figure 5**) varied with species (p < 0.001), but we did not observe a significant effect of time (p = 0.174) or heating (p = 0.211), indicating that silica losses tracked mass losses over time.

### Soil Solution and Stream Water DSi

**Figure 6:**
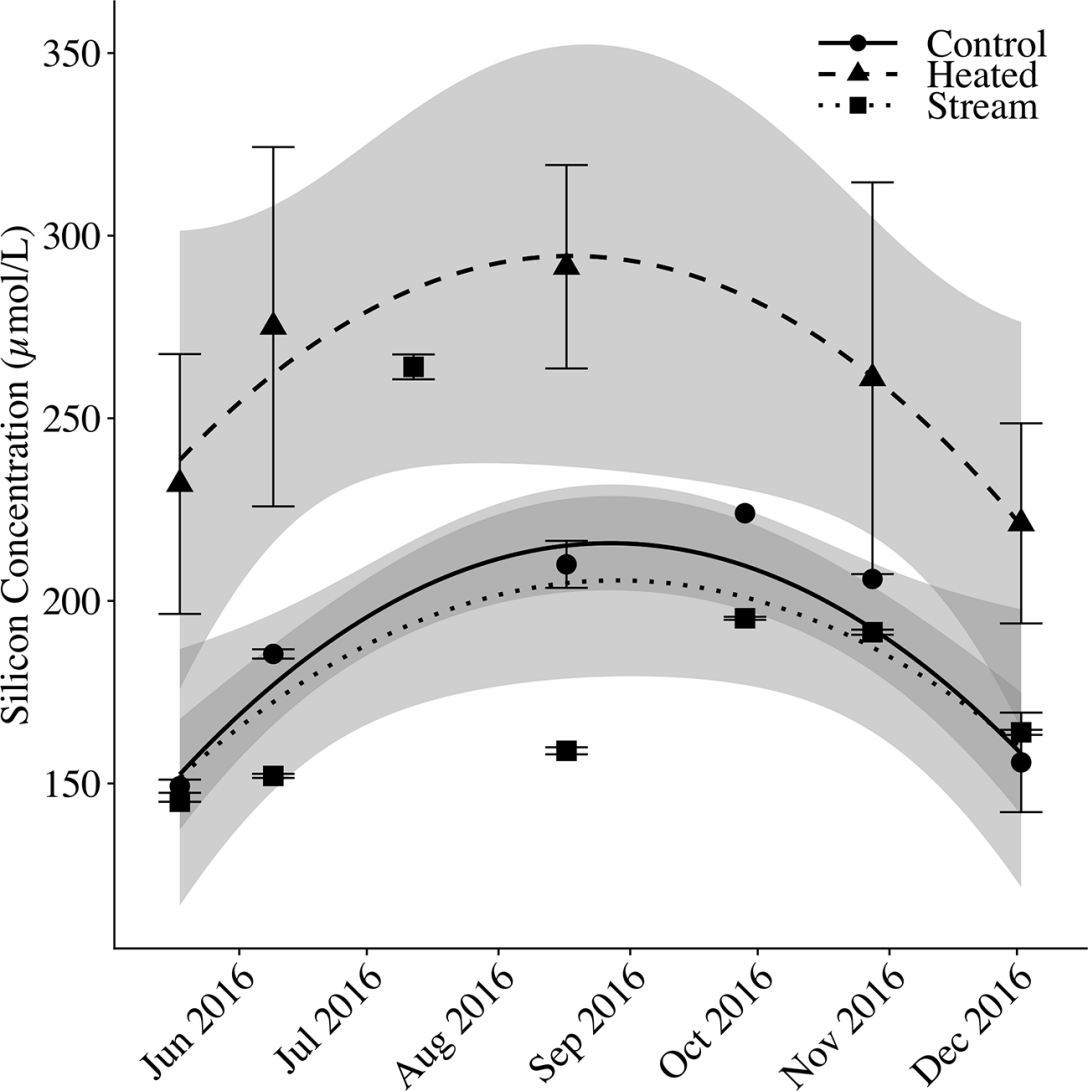
Soil solution and streamwater silicon over time. Mean silicon concentrations (μmol Si/L) are shown for control plot soil solution, heated plot soil solution, and stream water. For soil solution, silicon concentrations are measured as dissolved silica (DSi) only. For stream water, silicon concentration was measured as DSi plus suspended BSi to account for in-stream biological Si uptake. Error bars represent standard error of the mean. Fitted lines with shaded error bounds are second-degree polynomial regression models and 95% confidence intervals.

Mean soil solution DSi in the control plot and stream water DSi+BSi tracked closely with one another, whereas mean soil solution DSi in the heated plot was elevated above the other two pools (**Figure 6**). Across sampling dates, mean stream DSi+BSi concentrations were 181.53 ± 8.51 μM (mean DSi = 167.47 ± 7.64 μM; mean BSi = 14.06 ± 1.50 μM). Mean control plot soil solution DSi was 182.45 ± 7.18 μM, whereas mean heated plot soil solution DSi was 253.37 ± 15.57 μM, 39% greater relative to the control. There was a seasonal pattern to porewater and stream silica concentrations, with a peak in concentrations in August. The variation between sampling dates (p < 0.001) and silica pools (p < 0.001) were significant. The heated plot concentrations differed significantly from the control (p < 0.001) and the stream (p < 0.001), whereas the control plot soil solution was not significantly different than the stream water (p = 0.244).

## Discussion

This is the first study, to our knowledge, to estimate the effect of soil warming on the biogeochemical cycling of silica in a temperate forest, highlighting the impacts of long-term experimental manipulation on forest silica dynamics. We find evidence supporting our three hypotheses: first, soil warming increased net tree silica uptake, due to elevated biomass production and relatively constant leaf tissue silica concentrations. This increase in plant silica uptake was balanced by the increased return of BSi to the soil through litterfall. Second, soil warming led to an acceleration of silica release from decomposing litter: warming increased litter decomposition rates, and soil solution DSi concentrations were elevated in the heated plots. Third, these changes had no net effect on soil BSi stocks over time, likely due to silica inputs to the soil pool (i.e., litterfall and decay) balancing outputs (i.e., silica uptake by vegetation). Together, these data indicate faster internal silica cycling in temperate forests with warming. Below, we identify the likely mechanisms driving the observed changes.

### Soil Warming Effects on Plant BSi Production and Return Through Litterfall

We hypothesized that we would see an increase in plant silica uptake due to increases in productivity, which has been observed for other elements, such as carbon and nitrogen with warming (Melillo et al., 2011; Butler et al., 2012), as well as for silica under free-air CO_2_ enrichment (Fulweiler et al., 2015). We also expected to see differences in leaf concentration between species and possibly changes in concentrations or canopy storage over time as a result of long-term soil warming.

In this study, we did find significant differences in leaf BSi concentrations between species, with red maple consistently exhibiting silica concentrations two- to three-fold higher than those of red oak under ambient and warmed conditions. Both maple and oak foliar BSi concentrations were within the range previously reported. Fulweiler et al. (2015) measured 1.06% ± 0.12% BSi in red maple, and Clymans et al. (2016) measured 1.24% ± 0.42% BSi in sugar maple. Geis (1973) measured 0.327% BSi in red oak leaves, and Hodson et al. (2005) estimated 0.37% ± 0.01% BSi by in red oak leaves in a meta-analysis. We also observed significantly higher silica concentrations in fresh leaf litter compared to green foliage, consistent with the previous observations that leaf litter is enriched in silica compared to green leaf tissues (Lovering, 1959; Geis, 1973).

We found no difference in green leaf tissue BSi concentrations or leaf litter tissue BSi concentrations between the control and heated plot. However, we measured a 29% increase in litter production with warming, resulting in greater total litter BSi production.

This increase in plant productivity was within the expected range. Melillo et al. (2011) reported a 45% increase in vegetative carbon storage over the first 7 years of this soil-warming experiment, as measured by radial growth, and attribute the productivity enhancement to warming-driven increases in available nitrogen. The discrepancy of 16% between increases in litter production versus radial growth could be explained by many factors, such as mixing of litterfall between plots or differential impacts of warming on leaf and wood productivity.

The increase in litter mass was greater for red oak (31%) than that for red maple (19%). Year-to-year variation in the magnitude of litter productivity in the control and heated values closely tracked each other, indicating that local climate, rather than the warming treatment, drove inter-annual differences. Litterfall mass increased in both plots after 2006; however, this increase is likely a result of the aforementioned change in litterfall sampling procedures. Regardless, heated plot litter production was greater relative to the control in all years for red oak, and in all but 2 years for red maple.

We found a significant effect of sampling month (June through August) on green leaf silica concentrations, with the highest foliar silica concentrations observed in samples collected in August During each year of this study, foliar samples were collected during only 1 month of the growing season, making it difficult to distinguish between effects of phenology (early vs. late growing season) versus inter-annual variability or long-term changes on leaf silica concentrations. However, we find phenology to be the more likely explanation, as plants have been shown to continually accumulate silica in leaves throughout the growing season (Struyf et al., 2005; Carey and Fulweiler, 2013). Plant silica accumulation is driven by transpiration, and silica is immobile in plants after bio-silicification, leading to increased silica concentrations in older plant tissues (Ma and Yamaji, 2006). Moreover, we observed no trend across years in silica concentrations in fresh litter, which was collected at consistent times each year.

Because pre-treatment samples were not collected for green leaves or litter, we could not apply a correction factor to these data. However, given that we found no treatment effect on green leaf or litter BSi, we expect that pre-treatment correction would have been unlikely to affect our results.

#### Scaling to Canopy BSi Production

To obtain total canopy BSi production by red maple and red oak, we multiplied annual mean species concentrations in each plot by the mass of each species’ litter in each plot. For years in which we did not measure foliar BSi concentrations, we used the mean concentration for the species × plot across all years analyzed. We used data only from years in which litter was collected in laundry baskets to eliminate discrepancy between methods. We estimate that 20.2-kg BSi ha^−1^ year^−1^ is fixed in the control plot canopy by red oak and red maple combined, and 27.2 kg BSi ha^−1^ year^−1^ fixed in the heated plot canopy by red oak and red maple combined. This constitutes an increase of 35% of canopy silica fixation in the heated plot relative to the control, and consequently, a 35% increase in the fine litterfall flux of BSi to the forest floor. Our data thus support our first hypothesis: due to an increase in tree productivity at constant foliar silica concentrations, soil warming increased tree silica uptake, and release through litterfall.

**Figure 7:**
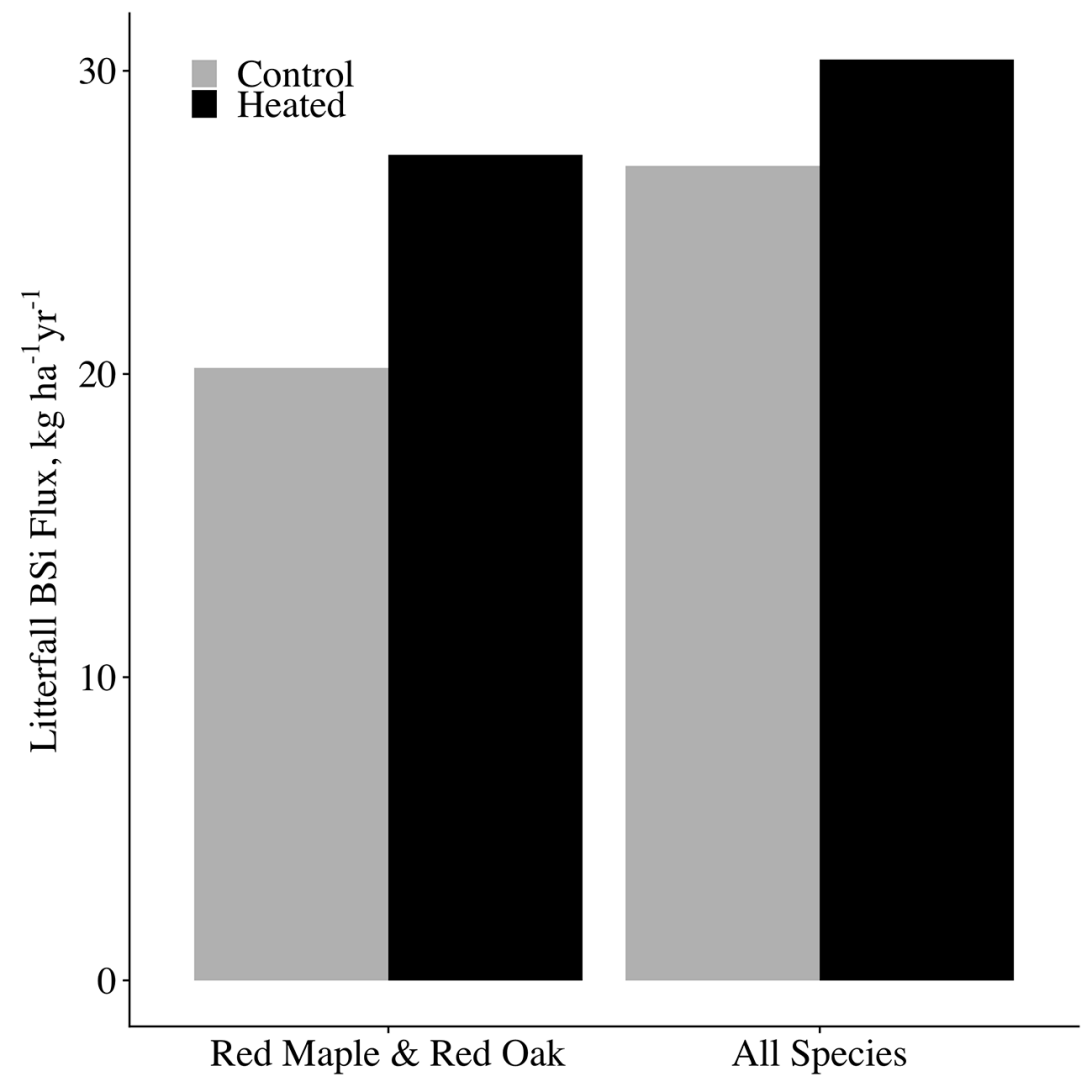
Annual canopy BSi production and release. A comparison between control and heated plot annual BSi uptake (and thus release through fine litterfall). BSi fluxes for each plot are shown for the species analyzed in this study only (red maple and red oak), as well as for our estimated of all species combined in each plot.

We took our measurements of red oak and red maple canopy BSi production and estimated concentrations for the remaining species using literature values (**Supplementary Table 3**) to estimate total canopy BSi production for each plot. The remaining species (making up a combined 29% of basal area) consisted of: *Acer pensylvanicum*, *Acer saccharum*, *Betula* sp., *Castanea dentata*, *Fagus grandifolia*, *Fraxinus americana*, *Populus grandidentata*, *Prunus serotina*, and *Quercus alba*. For *A. pennsylvanicum, C. dentata*, *F. grandifolia*, and *P. grandidentata*, species-specific concentrations were not available, so we used the mean of concentrations of the smallest available taxonomic classification containing each species. The published concentrations were for live biomass, so to obtain estimates for leaf litter concentrations, we applied an adjustment factor that equaled the mean of all leaf litter BSi concentrations measured in our study divided by the mean of all green foliage BSi (adjustment factor = 1.48). Given that we saw no effect of heating on red maple or red oak leaf BSi concentrations, we assumed the same to be true for the other species. We then multiplied our estimated litter BSi concentrations by mean litterfall mass per area for each species × plot, and took the sum of these values all species for each plot to obtain total litter BSi masses per plot. For all species combined, we estimate that soil warming increased canopy BSi production from 26.9-kg BSi ha^−1^ in the control plot to 30.4-kg BSi ha^−1^ in the heated plot (**Figure 7**). While we could not measure woody biomass silica concentrations for this study, we expect that the increase in woody biomass growth in the heated plot would likely further increase plant silica uptake and storage.

It should be noted that because the soil warming experiment consists of a single control plot and single heated plot, we cannot report error bounds for our estimates of plot-level phenomena. However, our estimates for canopy BSi production and return through litterfall are within the range of those previously reported for temperate forests: Cornelis et al. (2010a) report 4.5 to 90.3 kg BSi ha^−1^ in litterfall among a range of temperate forest types. Sommer et al. (2013) and Clymans et al. (2016) calculate overall uptake rates of 35 kg BSi ha^−1^ and 39 kg BSi ha^−1^, respectively, in similar deciduous forests.

It should be further noted that the experiment in our study involved soil warming only and should not be perceived as a simulation of whole-ecosystem climate change. In previous ecosystem-scale experiments, free-air CO_2_ enrichment (200 ppm) resulted in 20% increase in tree BSi uptake at the Duke Forest (Fulweiler et al., 2015), and soil freezing induced by snowpack manipulation resulted in a 28% decrease in sugar maple fine root Si uptake (Maguire et al., 2017). Interactions among these and other dimensions of global change may interact with soil warming in additive, synergistic, or antagonistic ways (Templer et al., 2017). Furthermore, given that the increase in silica uptake observed here depends on a nitrogen-mediated productivity enhancement, we note that soil warming could lead to contrasting or no effects of soil warming on silica cycling outside nitrogen-limited ecosystems. Understanding interactions between the biogeochemical cycles of multiple elements may be important for determining future alterations to terrestrial silica cycling.

### Soil Warming Effects on Silica Release From Decaying Litter and Retention in Soil

In our decomposition experiment, we found an acceleration of litter decay rates with soil warming for red maple, red oak, and birch leaves. The acceleration of litter decomposition in the heated plot was of the same order of magnitude as the increase in litterfall inputs. For each of the species we studied, there was no effect of warming on litter silica concentration over the course of decomposition, indicating that loss of BSi from decomposing litter tracked total biomass loss during decay, and that silica losses from decomposing litter were likewise accelerated by warming. This confirmed our second hypothesis: the increase in litterfall BSi inputs, together with the increased rate of silica losses from decomposing litter, suggests an increase in magnitude and rate of silica release from decaying biomass.

Both the relative decay rates among species and magnitude of warming-driven decay acceleration were in line with previously published decomposition studies. For example, a previous decomposition study in the same experimental plots found that 5°C of soil warming resulted in a 50% increase in decay rate constant for small red maple debris, and a 32% increase for small red oak debris (Berbeco et al., 2012), quite similar to the increases we observed of 47% increase for red maple leaves and 40% for red oak leaves.

In some cases, leaves (Eleuterius and Lanning, 1987) and coarse woody debris (Clymans et al., 2016) have been shown to exhibit preferential silica retention over decomposition. We note here that our litterbag incubation times were relatively brief and probably represented primarily decomposition of relatively labile carbon fractions. A longer-term incubation (and over-winter measurements) would likely have revealed slower overall decay rates (Harmon et al., 2009), and possibly different patterns in carbon-silica coupling.

Despite the increase in litterfall BSi flux to soils, we found no change in soil BSi concentrations over time in the organic or mineral layers between the control and heated plot. This again highlights faster internal recycling of BSi with warming, but no changes in net silica retention. Similar to concentrations, we saw no change in the overall stocks of BSi in soils with warming. However, soil horizon depths and bulk density were not measured so were assumed to be consistent over time. Further, due to the large stocks of BSi in the soils, detecting a change in BSi concentrations may take longer than 15 years. Nonetheless, we observe no measurable effect of warming on soil BSi concentrations or stocks over the duration of this experiment, as predicted in our third hypothesis.

While soil BSi appeared unchanged, DSi was elevated in the soil solution heated plots. The DSi concentrations we observed were within the typical range for forest soils (100–600 µM; Cornelis et al., 2010b), and the increase in soil solution DSi concentrations we observed in the heated plot reveals a probable impact of faster BSi decomposition in heated plots and a proximate source for the additional silica taken up by plants. This also supports the lack of change in soil BSi with warming, as increased inputs from litterfall appear balanced in part by increased dissolution and movement of Si from the soil BSi pool to the soil solution DSi pool.

### Connections to the Global Terrestrial Silica Cycle

In our study, we found that heating increased the fluxes of plant silica uptake and release through litterfall, with no net effect on soil silica stocks. There are two possible explanations for these trends. First, it is possible that enhanced plant silica uptake was balanced with enhanced BSi dissolution/release from decomposition, indicating accelerated internal Si recycling through the plant soil system, without changes to the weathering or leaching fluxes. Second, it is also possible that enhanced plant uptake exceeded the enhanced BSi dissolution rates, but increased mineral Si weathering inputs and/or reductions in leaching losses maintained the constant soil BSi stocks. In this case, an imbalance between changes to weathering and leaching fluxes could lead to enhancement or reduction of Si export to coastal receiving waters, with potential effects on marine primary productivity by diatoms and other silica-requiring species (Bristow et al., 2017).

The tight coupling between DSi observed in control plot soil solution and stream water in this study suggests to us that soil solution DSi is a dominant control on stream DSi in this system. Given that soil solution DSi was substantially elevated in the heated plot, we think it is likely that at least some portion of that additional DSi would be leached and delivered to streams. We also suspect that weathering inputs to soil DSi are increased by warming, as mineral silicate weathering typically increases with temperature as a result of reaction kinetics (Velbel, 1993; Brady and Carroll, 1994; White and Blum, 1995). Plants also influence mineral weathering through the physical and chemical alteration of soils (Lovering, 1959; Drever, 1994; Berner, 1997; Porder, 2019); thus, NPP enhancements may potentially lead to weathering increases in certain ecosystems (Kelly et al., 1998; Brault et al., 2017). In our study, this could lead to greater weathering inputs and leaching outputs of silica with warming, in addition to our observed enhancement of internal silica recycling.

Overall, our results indicate that soil warming can accelerate the biogeochemical cycling of silica in forests and increase the magnitude of the terrestrial silica pump (i.e., the uptake of DSi by land plants; Carey and Fulweiler, 2012a). The impacts of such changes on net vegetative silica storage and silica export from terrestrial to marine systems remain unresolved, but are likely important over the long term.

## Summary and Conclusions

Our study indicates that the biogeochemical cycle of silica, like that of carbon, nitrogen, and other nutrients, can be altered by soil warming, and thus is likely to be affected by changes in global surface temperatures with climate change. We find that soil warming increases plant silica uptake as a result of increased overall productivity at constant tissue silica concentrations. We further find an increase in the magnitude and return of silica from plants to soil through litterfall and litter decay. We additionally find that soil BSi stocks remained constant over the 15-year duration of this study, indicating a balance of increased silica inputs and outputs from the soil BSi pool. Our results confirm that soil warming increases the extent of internal silica recycling within a temperate forest ecosystem, with potential implications for the global terrestrial silica pump, and land and ocean carbon cycling. These results underscore the need to further explore the interactions between geology and biology with climatic change to understand and predict future alterations to the global silica cycle.

## Data Availability

The datasets generated for this study are available on request to the corresponding author.

## Author Contributions

JG, JC, and JT conceived of the project. JG collected silica data with support from JC, JT, RF, and AK. JM conceived of the soil warming experiment and JM and WW provided samples, long-term data, and critical insight from the experiment. JG analyzed data and led the writing of the manuscript, and all authors contributed to drafts and the final paper.

## Funding

This research was supported by the National Science Foundation (NSF PLR-1417763 to JT), the Geological Society of America (Stephen G. Pollock Undergraduate Research Grant to JG), the Institute at Brown for Environment and Society, and the Marine Biological Laboratory. Sample analysis and Fulweiler’s involvement were supported by Boston University and a Bullard Fellowship from Harvard University. The soil warming experiment was supported by the National Science Foundation (DEB-0620443) and Department of Energy (DE-FC02-06-ER641577 and DE-SC0005421).

## Conflict of Interest Statement

The authors declare that the research was conducted in the absence of any commercial or financial relationships that could be construed as a potential conflict of interest.
