# Supplementary material for: Soil Warming Accelerates Biogeochemical Silica Cycling in a Temperate Forest
Source: Front Plant Sci. 2019 Sep 11;10:1097. doi: 10.3389/fpls.2019.01097 (PMC6749086; doi:10.3389/fpls.2019.01097)
Supplement: Supplementary file 1 [file Table_1.docx]

Supplementary Material

Supplementary Table 1: Soil BSi concentrations by year, raw and pretreatment-corrected data. In the pre-treatment (2002) soil samples, we found higher mean BSi concentrations in the heated plot compared to the control plot (Table 1). While the difference between means (n=3 for each plot x layer x year), was not statistically significant, it was substantial (17% difference for organic and 21% for mineral soil. As such, we present the data in our paper after scaling the heated treatment soil data by a pre-treatment correction factors, which we calculated according to the methods described by Meillilo et al. (2011). Specifically, the pre-treatment correction factor scales the pre-treatment heated plot BSi concentration to equal the pre-treatment control plot BSi concentration in order to account for pre-existing site differences. We calculated correction factors for mineral and organic soil layers using the pre-treatment (2002) data, and applied the correction factors to the treatment period data (2005, 2010, 2015). We report data here both with and without the pretreatment correction. BSi concentrations are reported as percent dry weight biogenic silica (SiO_2_).

| **Layer** | **Plot** | **Correction Applied** | **% Dry wt BSi (2002)** | **% Dry wt BSi (2005)** | **% Dry wt BSi (2010)** | **% Dry wt BSi (2015)** |
| --- | --- | --- | --- | --- | --- | --- |
| Mineral | Control | No | 0.98 ± 0.2 | 0.59 ± 0.09 | 0.79 ± 0.08 | 0.73 ± 0.05 |
|  | Heated | No | 1.19 ± 0.15 | 0.95 ± 0.07 | 0.82 ± 0.11 | 0.93 ± 0.04 |
|  | Heated | Yes |  | 0.78 ± 0.05 | 0.67 ± 0.09 | 0.77 ± 0.03 |
| Organic | Control | No | 1.03 ± 0.08 | 0.77 ± 0.06 | 1.08 ± 0.11 | 0.84 ± 0.12 |
|  | Heated | No | 1.2 ± 0.10 | 1 ± 0.07 | 1.32 ± 0.05 | 0.99 ± 0.11 |
|  | Heated | Yes |  | 0.86 ± 0.06 | 1.13 ± 0.05 | 0.84 ± 0.10 |

Supplementary Table 2: Soil BSi stocks. Soil BSi stocks were calculated for the top 10 cm in each plot, and the data reported here are means across all samples analyzed from all years during experimental treatment. Both raw values and pretreatment-corrected values are reported here.

| **Layer** | **Treatment** | **BSi (kg ha ^-1^),**  **Uncorrected** | **BSi (kg ha ^-1^),**  **Pre-Treatment Corrected** |
| --- | --- | --- | --- |
| Organic | Control | 464.44 ± 35.83 | 464.44 ± 35.83 |
|  | Heated | 571.61 ± 35.28 | 488.01 ± 30.12 |
| Mineral | Control | 4723.97 ± 320.07 | 4723.97 ± 320.07 |
|  | Heated | 6030.09 ± 292.47 | 4957.93 ± 240.47 |

**Supplementary Table 3.** Average litterfall composition at the Barre Woods soil experiment between 2006-2016, and BSi concentrations used in this study to estimate total plot-level canopy BSi fixation. Litter mass percent composition was calculated as total dry litter mass for each species divided by total litter mass for all species combined within each plot. Litter BSi concentrations were measured directly in this study in *Quercus rubra* and *Acer rubrum*. For all other species present in the plots, literature values for green leaves were used and scaled by the ratio of litter BSi concentration to green leaf concentration measured in our study (scaling factor=1.48). When species-specific values were not available in the literature, we used the mean value for the smallest taxonomic classification containing that species. The final BSi concentrations which we used (directly measured values or scaled literature values) are reported here. The source of literature values is also reported, along with the taxonomic unit used.

| **Species** | **Control Plot Percent Composition (Dry Mass)** | **Heated Plot Percent Composition (Dry Mass)** | **Litter Percent Dry Weight BSi** | **BSi Data Source** | **Smallest Taxonomic Unit Available** |
| --- | --- | --- | --- | --- | --- |
| *Quercus rubra* | 57.9 | 73.7 | 0.56 | Measured directly in this study | Species |
| *Acer rubrum* | 14.2 | 16.5 | 1.83 | Measured directly in this study | Species |
| *Betula sp.* | 12.5 | 2.7 | 0.98 | Hodson et al., 2005 | Genus (*Betula*) |
| *Fraxinus americana* | 11.6 | 3.3 | 0.15 | Hodson et al., 2005 | Species |
| *Acer saccharum* | 1.9 | 1.4 | 1.66 | Hodson et al., 2005 | Species |
| *Populus grandidentata* | 1.2 | 0 | 1.22 | Hodson et al., 2005 | Genus (*Populus*) |
| *Acer pennsylvanicum* | 0.2 | 0.8 | 1.06 | Hodson et al., 2005 | Genus (*Acer*) |
| *Castanea dentata* | 0.1 | 0.8 | 1.58 | Hodson et al., 2005 | Family (*Fagaceae*) |
| *Fagus grandifolia* | 0.1 | 0 | 2.18 | Clymans et al., 2016 | Species |
| *Prunus serotina* | 0.1 | 0.3 | 0.41 | Hodson et al., 2005 | Species |
| *Quercus alba* | 0 | 0.1 | 1.00 | Hodson et al., 2005 | Species |
| Unknown species | 0 | 0.3 | 1.15 | Used mean value of all known species in this study | N/A |
